# Trimethoxy-benzaldehyde levofloxacin hydrazone inducing the growth arrest and apoptosis of human hepatocarcinoma cells

**DOI:** 10.1186/1475-2867-13-67

**Published:** 2013-07-02

**Authors:** Jin-ping Sun, Zhen-yu Shi, Shi-meng Liu, Yu-hua Kang, Guo-qiang Hu, Chao-shen Huangfu, Jin-bo Deng, Bin Liu

**Affiliations:** 1 College of Nursing, Institute of Neurobiology, Henan University, Kaifeng, China; 2 Huaihe Clinical College, Henan University, Kaifeng, China; 3 Institute of Chemical Biology, Henan University, Kaifeng, China

**Keywords:** Fluoroquinolone derivatives, Hepatocarcinoma cell line, DNA topoisomerase II, Mitochondrial dysfunction, Apoptosis

## Abstract

**Background:**

In order to search for new structural modification strategies on fluoroquinolones, we have designed and synthesized a series of fluoroquinolone derivatives by linking various hydrazine compounds to the C-3 carboxyl group of levofloxacin and assessed their anticancer activities. Several novel levofloxacin derivatives displayed potent cytotoxicity against the tested cancer cell lines in vitro. In the present study, we investigated the effect of 1-Cyclopropyl-6-fluoro-4-oxo-7- piperazin-1, 4-dihydro- quinoline- 3-carboxylic acid benzo [1,3] dioxol-5- ylmethylene- hydrazide (QNT11) on the apoptosis of human hepatocarcinoma cells in vitro.

**Methods:**

The inhibition effects of QNT11 on cell proliferation were examined by MTT assay. Cell apoptosis was determined by TUNEL and DNA agarose gel electrophoresis method. The topoisomerase ΙΙ activity was measured by agarose gel electrophoresis using Plasmid pBR322 DNA as the substrate. Cell cycle progression was analyzed using flow cytometry in conjunction with ethanol fixation and propidium iodide staining. Mitochondrial membrane potential (△ψm) was measured by high content screening image system. The caspase-9, caspase-8, caspase-3, Bcl-2, Bax, CDK1, Cyclin B1and cytochrome c protein expressions were detected by Western blot analysis.

**Results:**

QNT11 showed selective cytotoxicity against Hep3B, SMMC-7721, MCF-7 and HCT-8 cell lines with IC_50_ values of 2.21 μM, 2.38 μM, 3.17 μM and 2.79 μM, respectively. In contrast, QNT11 had weak cytotoxicity against mouse bone marrow mesenchymal stem cells (BMSCs) with IC_50_ value of 7.46 μM. Treatment of Hep3B cells with different concentrations of QNT11 increased the percentage of the apoptosis cells significantly, and agarose gel electrophoresis revealed the ladder DNA bands typical of apoptotic cells, with a decrease in the mitochondrial membrane potential. Compared to the control group, QNT11 could influence the DNA topoisomerase IIactivity and inhibit the religation of DNA strands, thus keeping the DNA in fragments. There was a significant increase of cytochrome c in the cytosol after 24 h of treatment with QNT11 and a decrease in the mitochondrial compartment. Observed changes in cell cycle distribution by QNT11 treated might be caused by insufficient preparation for G_2_/M transition. In addition, QNT11 increased the protein expression of Bax, caspase-9, caspase-8, caspase-3, as well as the cleaved activated forms of caspase-9, caspase-8 and caspase-3 significantly, whereas the expression of Bcl-2 decreased.

**Conclusions:**

Our results showed that QNT11 as a fluoroquinolone derivative exerted potent and selectively anticancer activity through the mechanism of eukaryotic topoisomerase II poisoning. The growth inhibition was in large part mediated via apoptosis-associated mitochondrial dysfunction and regulation of Bcl-2 signaling pathways.

## Background

Antibacterial fluoroquinolone is a very important family of antibacterial drugs that are widely prescribed for the treatment of infections in humans [1]. According to the pharmacological mechanisms elucidated in numerous reports, antibacterial fluoroquinolone corrupts the activities of prokaryotic type II topoisomerase, DNA gyrase, and induces them to kill cells by generating high levels of double-stranded DNA breaks. DNA gyrase modulates the topological state of the genetic material by passing an intact DNA helix through a transient double-stranded break to generate a separate DNA segment [2]. Like bacterial cells, eukaryotic species also require a type II topoisomerase, known as topoisomeraseII, for viability. Comparing the known sequences of type II topoisomerases of bacteria and mammals, the sequences around activated tyrosine residues appear to have common homology [3]. The mechanisms responsible for cell killing by antitumor fluoroquinolones also appear to be similar to that of quinolone antibacterial agents [4]. Although 100-fold more sensitive to prokaryotic DNA gyrase, fluoroquinolones also have some inhibitory effects on mammalian DNA topoisomerase II. In addition, their mode of action is similar to that of anthracycline derivatives (such as doxorubicin, amsacrine, mitoxantrone), epipodophyllotoxin derivatives (such as etoposide), and actinomycin D (a class of polypeptide antibiotics isolated from *Streptomyces*). For this reason, antibacterial fluoroquinolones have been shown to have cytotoxic activity against cancer cells [5,6], thus representing a potentially important source of new anticancer agents.

Recently, the development of antitumor agents out of antibacterial fluoroquinolones has attracted much attention based on the mechanistic similarities and sequence homologies of the targeting eukaryotic topoisomerases [7]. However, many antitumor fluoroquinolones were modified from clinical antibacterial fluoroquinolones with regard to the nitrogen-containing ring, such as piperazine, on the 7-position and (or) the 2-position of fluoroquinolone scaffold [8,9]. In addition, a few modifications for the carboxylic group at the 3-position were reported [10]. Indeed, it doesn’t seem necessary for an antitumor fluoroquinolone to retain the carboxylic group; fluroquinolones with a fused heterocyclic ring as an isostere of the carboxylic group showed good anticancer activity as well as excellent water solubility [11].

To search for new structural modification strategies on antibacterial fluoroquinolones, we have designed and synthesized a series of fluoroquinolone derivatives by linking various hydrazine compounds to the C-3 carboxyl group of ofloxacin or ciprofloxacin and assessed their anticancer activities. Several novel levofloxacin derivatives displayed potent cytotoxicity against the tested cancer cell lines in vitro, where the IC_50_ values of the compounds reached micromolar concentration [12]. The IC_50_ of QNT11 (1-Cyclopropyl-6-fluoro-4-oxo-7-piperazin-1, 4-dihydro- quinoline- 3-carboxylic acid benzo [1,3] dioxol-5-ylmethylene- hydrazide) was the lowest among the levofloxacin derivatives. Therefore we investigated the growth inhibitory effects and the molecular mechanisms of QNT11 in human hepatocarcinoma cells *in vitro*. We found that QNT11 showed potent cytotoxicity against Hep3B cells with an IC_50_ value of 2.21 μM. We also investigated the signal pathways of QNT11-induced apoptosis.

## Methods

### Chemicals

**Figure 1:**
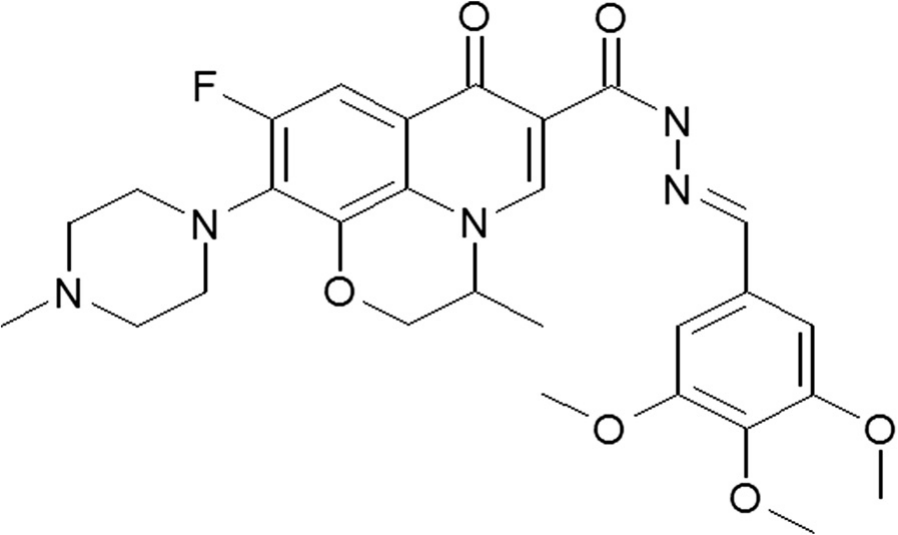
**Chemical structure of 1-Cyclopropyl-6-fluoro-4-oxo-7-piperazin-1,4-dihydro-quinoline-3-carboxylic acid benzo**[1,3]**dioxol**-**5**-**ylmethylene**- **hydrazide** (**QNT11**).

Trimethoxy-benzaldehyde levofloxacin hydrazone (QNT11) was synthesized at the Institute of Chemistry and Biology at Henan University. The purity was more than 98% by HPLC analysis. The compound was dissolved in dimethyl sulfoxide (DMSO). Its structure is illustrated in Figure 1.

### Cell culture

Human hepatocarcinoma cells (Hep3B2.1-7 and SMMC-7721), human breast adenocarcinoma cells (MCF-7), and human colon adenocarcinoma cells (HCT-8) from the Institute of Cytology, Chinese Academy of Sciences (Shanghai, China), were cultured in DMEM medium (Gibco BRL, USA) supplemented with 10% (v/v) heat-inactivated fetal bovine serum, 100 IU/ml penicillin and 100 μg/ml streptomycin. The cells were maintained in 5% CO_2_, at 37°C until reaching approximately 50%-70% confluence and were then treated with different amounts of chemicals as indicated. DMSO alone was used as the vehicle control.

### Isolation of BMSCs

Bone marrow from the tibias and femurs of approximately 8 week old mice was flushed with DMEM. The marrow cells were dissociated by passage through a 25-gauge needle. The cell suspension was centrifuged at 1000 rpm for 5 min, re-suspended in DMEM medium supplemented with 10% (v/v) fetal bovine serum, 100 IU/ml penicillin and 100 μg/ml streptomycin, and plated in 100 mm culture dishes with supplemented DMEM and maintained in 5% CO_2_, at 37°C for 24 h, the medium containing the non-adherent cells was removed, and the adherent cells were gently washed 2 times with PBS to reduce the degree of hematopoietic lineage cell contamination. The cells were cultured in DMEM for 3–4 weeks, until they reached 50-70% confluence.

### MTT assay

The cells were seeded at a density of 1 × l0^4^ cells/well in 96-well culture plates, and 24 h later they were treated with the indicated concentrations of QNT11 or levofloxacin. Control wells consisted of cells incubated with medium only. After 12, 24, 48 and 72 h of treatment, cells were incubated with 20 μl 3-(4,5-dimethylthiazol-2-yl) 2,5-diphenyltetrazolium bromide (MTT, Sigma, St Louis, MO, USA) at 5 mg/ml. After 4 h at 37°C, the supernatant was removed, and 150 μl DMSO was added. After the blue crystals were dissolved in DMSO, the optical density (OD) was then detected at a 570 nm wavelength using a 96-well multiscanner autoreader (Bio-Rad, USA). The following formula was used to determine the inhibition of cell proliferation: cell proliferation inhibited (%) = [1-(OD of the experimental samples/OD of the control)] × 100. The IC_50_ was the concentration that caused a 50% inhibition of cel1 proliferation.

### TUNEL assay

Hep3B cells (4 × l0^4^ cells/ml) were seeded in growth medium on the cover glass slides of 6-well plates for 24 h incubation. They were then treated with the indicated concentrations of QNT11 for 24 h. Control wells consisted of cells incubated with medium only. After that, cells were examined for apoptosis by terminal deoxynucleotidyl transferase-mediated dUTP nick-end labeling (TUNEL) assay (Promega, Madison, WI, USA), performed according to the manufacturer’s instructions. Cells were visualized and photographed using a fluorescent microscope (BX51, Olympus, Japan). At least five randomly chosen areas in every slide were used. Percent apoptosis was determined by counting the number of apoptotic cells and dividing by the total number of cells in the areas.

### DNA agarose gel electrophoresis

Hep3B cells were treated with media containing different concentrations of QNT11 for 24 h and were then washed twice with PBS. The chromosomal DNA was extracted with Apoptotic DNA Ladder Detection Kit (Beyotime, China) according to the manufacturer’s instructions. The DNA sample was incubated at 37°C for 30 min and electrophoresed on a 1% agarose gel containing 1 mg/ml ethidium bromide at 40 V/cm. Finally, the apoptotic DNA fragments were visualized under a UV transilluminator and photographed.

### Flow cytometry analysis

Hep3B cells (1 × 10^6^/ml) were washed twice with ice-cold PBS and then were re-suspended gently in 500 μl of ice-cold PBS. Thereafter, ice-cold 70% ethanol (4 ml) was added in a dropwise manner and cells were stored at 4°C for 12 h. After 12 h, cells were pelleted by centrifugation for 5 min. The supernatant was removed and cells were re-suspended in 500 μl of PBS containing propidium iodide (50 μg/ml) and incubated in dark conditions at 37°C before analysis by FACS (Calibur, Becton Dickinson).

### Topoisomerase II-mediated supercoiled pBR322 DNA relaxation assay

DNA topoisomeraseIIactivity was determined by the supercoiled pBR322 DNA relaxation assay [13]. The experiments were performed by incubating human topoisomeraseIIα (Sigma, St Louis, MO, USA) with 1 μg supercoiled pBR322 DNA in 5 μl relaxation buffer (200 mM Tris–HCl, pH 7.5, 340 mM KCl, 40 mM MgCl_2_, 20 mM DTT, 120 mg/L BSA, 5 mM EDTA, 4 mM ATP) under increasing concentrations of QNT11. In this experiment, etoposide (Sigma, St Louis, MO, USA), a known topoisomeraseIIpoison [14], was used as a positive control. Reactions were incubated at 37°C for 30 min and terminated by adding 20 μl 10% SDS, and 1μl protease K (1 × l0^4^ mg/L). Samples were subjected to electrophoresis in 1% agarose gels. DNA was then stained with 1 mg/ml ethidium bromide and photographed under a UV transilluminator.

### Estimate of mitochondrial membrane potential loss

Hep3B cells were treated with media containing different concentrations of QNT11 for 24 h and were then incubated with 0.5 mg/ml of the fluorescence probe JC-1 (5,5',6,6'-tetrachloro-l, l',3,3'- tetraethyl-benzimidazolcarbocyanine iodide, Beyotime, China) at 37°C for 20 min. The cells were washed twice thoroughly with buffer and incubated with 5 μg/ml. Hoechst 33258 for 10 min in the dark. After additional washing twice, the mitochondrial membrane potential (△ψm) was measured by high content screening (HCS) image system. (Thermo Fisher Scientific, USA).

### Western blot analysis

After treatments with different concentrations QNT11 for 24 h, Hep3B cells were lysed with ice-cold RIPA lysis buffer. Protein concentrations were determined using the Bradford method. After adjustment to a similar level of total protein concentration, samples were separated by 12% SDS–PAGE under reducing conditions and then transferred onto polyvinglidene fluoride (PVDF) membranes (Millipore). The membranes were blocked with 5% non-fat milk in TBST buffer (20 mM Tris–HCl, 137 mM NaCl, and 0.1% Tween 20, pH 8.0) for 1 h at room temperature prior to incubation with specific antibodies to caspase-9, caspase-8, caspase-3, Bcl-2, Bax, CDK1, CyclinB1, Cytochrome *c* or β-actin (all antibodies from Santa Cruz Biotechnology) overnight at 4°C. After washing and reaction with horseradish peroxidase conjugated anti-mouse IgG (Beijing Zhong Shan Golden Bridge Biological Technology CO, LTD), or anti- rabbit IgG (Beyotime, China) secondary antibodies for 1 h, the membranes were washed with TBST buffer three times and the proteins on the membrane were detected using an enhanced chemiluminescene substrate (ECL, Beyotime, China).

Cytochrome *c* release from mitochondria was evaluated by western blot analysis of cytosolic protein samples. Cytosolic and mitochondrial protein fractions were prepared using the cell mitochondria isolation kit (Beyotime, China).

### Statistical analyses

Data are presented as the mean ± standard deviation (SD) for the indicated number of independent experiments. Statistical significance was calculated using the *t*-test for paired samples. *P* < 0.05 was regarded as significant, and *P* < 0.01 as highly significant.

## Results

### QNT11 suppressed the growth of the cancer cells in vitro

**Figure 2:**
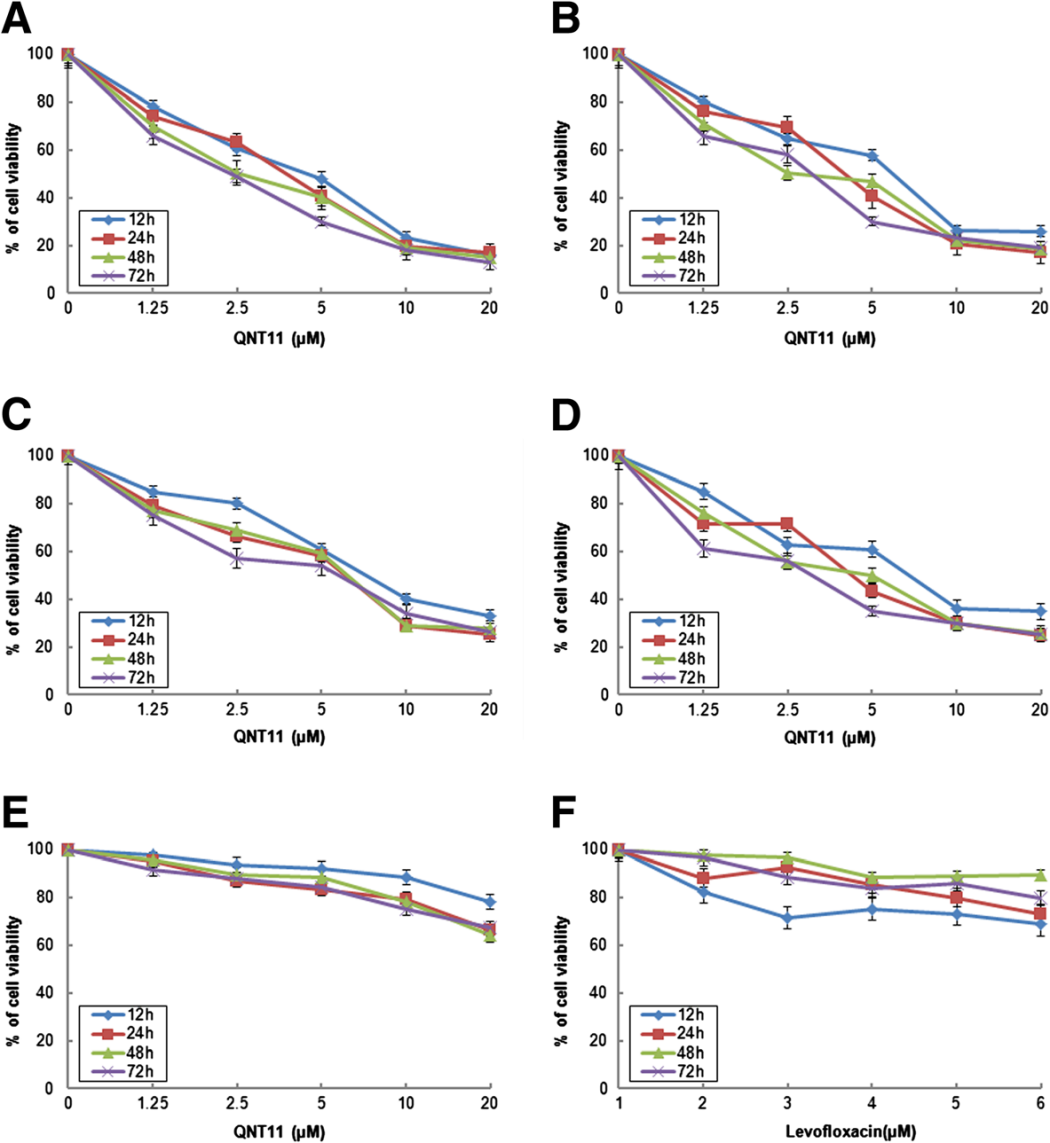
**Inhibition of cell viability by QNT11 and levofloxacin treatment in human cancer cells and BMSCs.** Hep3B cells **(A)**, SMMC-7721 cells **(B)**, MCF-7 cells **(C)**, HCT-8 cells **(D)** and BMSCs **(E)** were treated with various concentrations of QNT11 for 12–72 h. Hep3B cells were treated with various concentrations of Levofloxacin **(F)** for 12–72 h. Control cells were treated with the same volume of DMSO as a vehicle control (the final concentration of DMSO was below 0.1%). After treatment, cell viability was measured by MTT assay as described in Methods, and then calculated as a percentage of viability of the control cells. Data represent means ± SD of three independent measurements.

The cytotoxicity of QNT11 against cells was assessed using MTT cell viability assay. The cells were treated with various concentrations of QNT11 for 12,24, 48 and 72 h, resulting in a significant decrease in cell viability in a dose- and time-dependent manner (Figure 2). Within the four cancer cell lines used in this experiment, QNT11 was more effective against Hep3B cells. As shown in Figure 2A, the IC_50_ value for 12,24, 48 and 72 h treatment was 1.92 ± 0.19 μM (*r*^*2*^ = 0.9601), 2.21 ± 0.20 μM (*r*^*2*^ = 0.9679), 2.55 ± 0.25 μM (*r*^*2*^ = 0.9561) and 2.70 ± 0.22 μM (*r*^*2*^ = 0.9802) respectively. For SMMC-7721 cells, the IC_50_ value for 12, 24, 48 and 72 h treatment obtained was 2.16 ± 0.28 μM (*r*^2^ = 0.9109), 2.38 ± 0.22 μM (*r*^2^ = 0.9408), 2.68 ± 0.25 μM (*r*^*2*^ = 0.9427) and 3.06 ± 0.19 μM (*r*^2^ = 0.9264), respectively. For MCF-7 cells, the IC_50_ was 2.93 ± 0.23 μM (*r*^2^ = 0.9640), 3.17 ± 0.26 μM (*r*^2^ = 0.9212), 3.11 ± 0.32 μM (*r*^2^ = 0.9490) and 3.68 ± 0.25 μM (*r*^2^ = 0.9624) for 12, 24, 48 and 72 h treatment respectively. For HCT-8 cells, the IC_50_ value for12, 24, 48 and 72 h treatment obtained was 2.12 ± 0.22 μM (*r*^2^ = 0.9260), 2.79 ± 0.22 μM (*r*^2^ = 0.9547), 2.86 ± 0.25 μM (*r*^2^ = 0.9139) and 3.46 ± 0.32 μM (*r*^2^ = 0.9174), respectively. In contrast, QNT11 had weak cytotoxicity against BMSCs. The IC_50_ value for 12, 24, 48 and 72 h treatment was 8.13 ± 0.55 μM (*r*^*2*^ = 0.9574), 7.46 ± 0.50 μM (*r*^*2*^ = 0.9034), 7.90 ± 0.55 μM (*r*^*2*^ = 0.9414) and 12.05 ± 0.69 μM (*r*^*2*^ = 0.8993), respectively.

Levofloxacin had weak cytotoxicity against Hep3B cells. The IC_50_ value for 12, 24, 48 and 72 h treatment was 12.46 ± 0.41 μM (*r*^*2*^ = 0.6261), 10.99 ± 0.39 μM (*r*^*2*^ = 0.8016), 20.80 ± 0.58 μM (*r*^*2*^ = 0.7122) and 13.03 ± 0.46 μM (*r*^*2*^ = 0.8362), respectively.

### QNT11 Induced apoptosis of Hep3B cells

**Figure 3:**
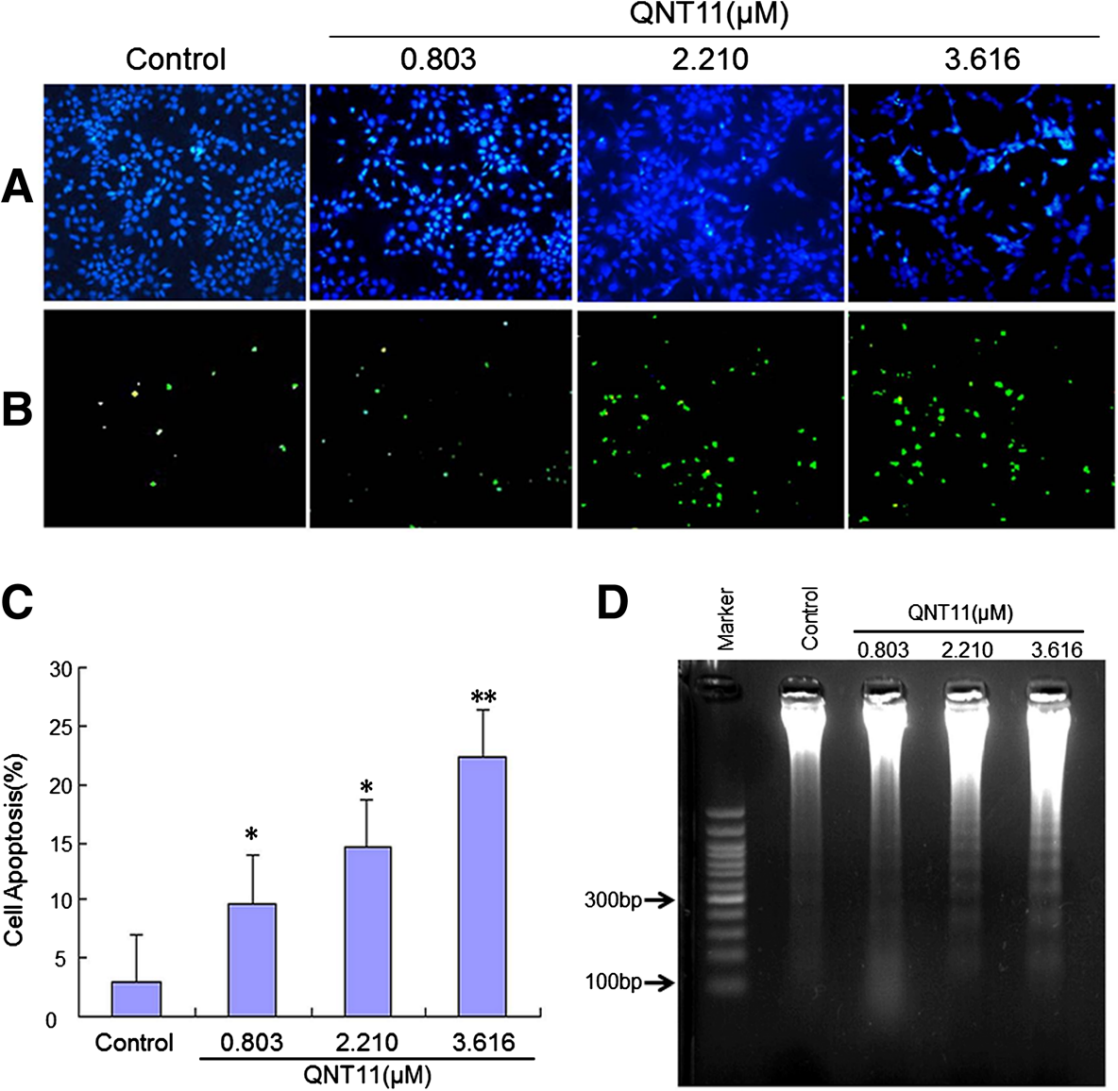
**QNT11 induces apoptosis in Hep3B cells.** Induction of apoptosis in Hep3B cells treated with QNT11 for 24 h was evaluated by TUNEL assay. Representative images were taken with nuclear stain DAPI, **(A)** and apoptosis stain TUNEL, **(B)** × 200. **(C)** Data are expressed as overall means ± SD from three independent experiments. Statistical significance was determined using the Student’s *t*-test (* *P* < 0.05, ** *P* < 0.01 vs control). **(D)** Hep3B cells treated with 0, 0.803 ( IC_30_ group), 2.210 (IC_50_ group) and 3.616 μM (IC_70_ group) QNT11 respectively for 24 h. DNA from 1 × 10^6^ cells was electrophoresed through 1% agarose gels and stained with 0.5 μg/ml ethidium bromide.

After treatment with various concentrations of QNT11 for 24 h, TUNEL assay was used to confirm the apoptotic effect of QNT11. 24 h incubation with QNT11 increased the percentage of apoptotic Hep3B cells in a concentration-dependent manner (Figure 3A,B and C).

The integrity of DNA was assessed by agarose gel electrophoresis. As shown in Figure 3D, 24 h incubation of Hep3B cells with 2.210 μM and 3.616 μM QNT11 elicited a characteristic DNA “ladder” bands indicative of apoptotic internucleosomal DNA fragmentation (about 180 ~ 200 bp).

### Effects of QNT11 on the catalytic activities of eukaryotic topoisomerase II

**Figure 4:**
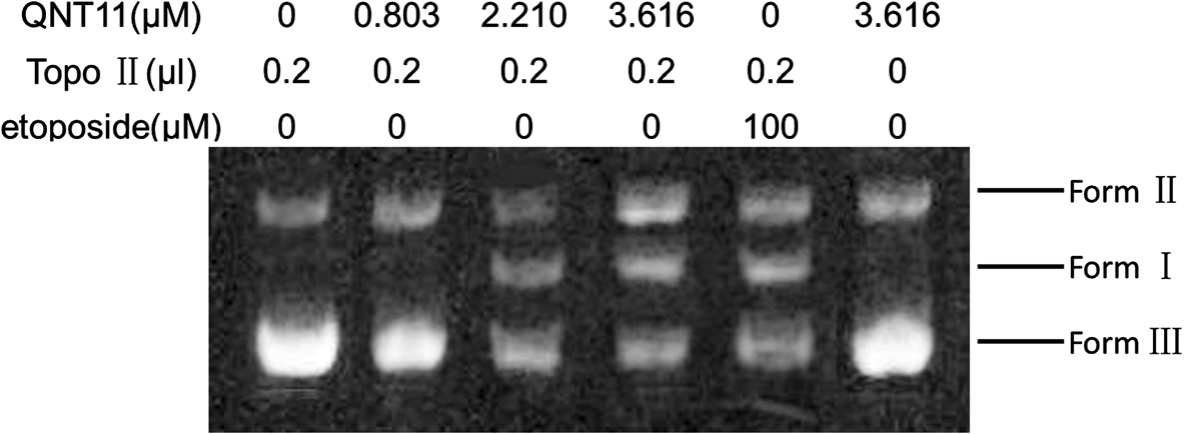
**The inhibition effects of QNT11 on DNA topoisomerase II activity.** Human topoisomeraseIIα was incubated with supercoiled pBR322 DNA in relaxation buffer under increasing concentrations of QNT11. Samples were subjected to electrophoresis in 1% agarose gels and then stained with ethidium bromide and photographed under a UV transilluminator.

In this experiment, the relative effects of QNT11 on double-stranded DNA cleavage/relegation in the presence of topoisomerase II were determined by agarose gel electrophoresis of treated-pBR322 DNA and nontreated-pBR322 DNA (Figure 4). QNT11 decreased the supercoiled DNA (Form I), while increasing nicked circular plasmid molecules (Form II) and linear molecules (Form III) in a dose-dependent manner. The effects of QNT11 on topoisomeraseII-mediated DNA cleavage/religation were similar to that of etoposide. This suggests that QNT11 increased topoisomerase II-mediated DNA breakages but inhibited DNA religation.

### Effects of QNT11 on the mitochondrial membrane potential

**Figure 5:**
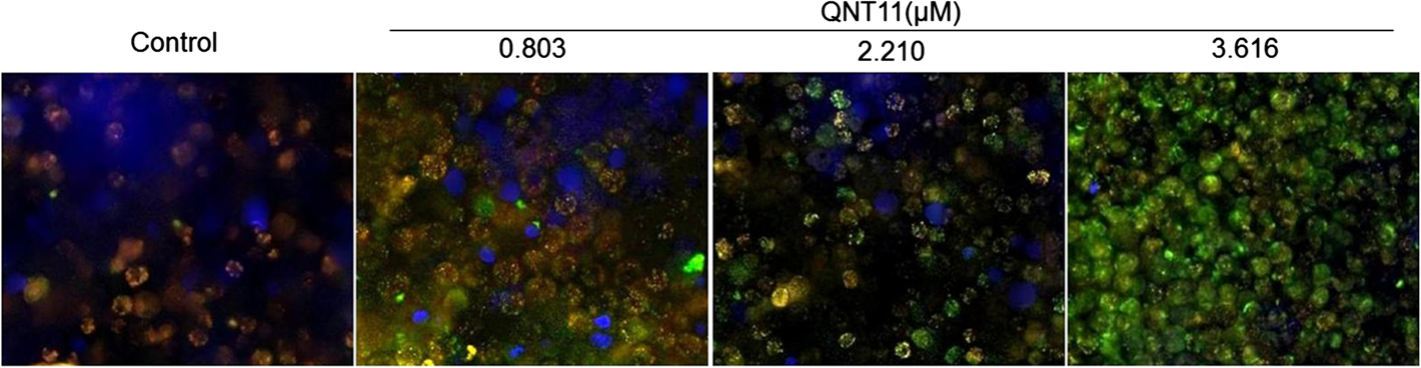
**Effects of QNT11 on the mitochondrial membrane potential of Hep3B cells HCS analysis after JC-1 staining to detect changes in the mitochondrial membrane potential of Hep3B cells Induced by QNT11.** Cells were treated with 0, 0.803 (IC_30_ group), 2.210 (IC_50_ group)and 3.616 μM (IC_70_ group) QNT11 respectively for 24 h. The mitochondrial membrane potential (△ψm) was measured by HCS image system.

Mitochondrial membrane potential (△ψm) was detected with the fluorescent probe JC-1, which exists predominantly in monomeric form in cells with depolarized mitochondria and displays green fluorescence at 490 nm. On the other hand, JC-1 primarily forms aggregates in cells with polarized mitochondria and shows reddish-orange fluorescence. The emission intensity ratio of the 545 nm and 595 nm peaks was used as a measure of the mitochondrial depolarization; a higher ratio indicated more depolarization. Hep3B cells with QNT11 treatment for 24 h exhibited green JC-1 fluorescence in a dose-dependent manner, which is consistent with a loss of △ψm (Figure 5). After treatment with QNT11 at 0.803 μM, 2.210 μM and 3.616 μM for 24 h, △ψm of the cells decreased (9.65 ± 4.26)%, (37.34 ± 3.98)% and (45.05 ± 3.18)%, respectively, as compared to the control (P < 0.05,T = 3.24).

### Effects of QNT11 on apoptotic protein expressions in Hep3B cells

**Figure 6:**
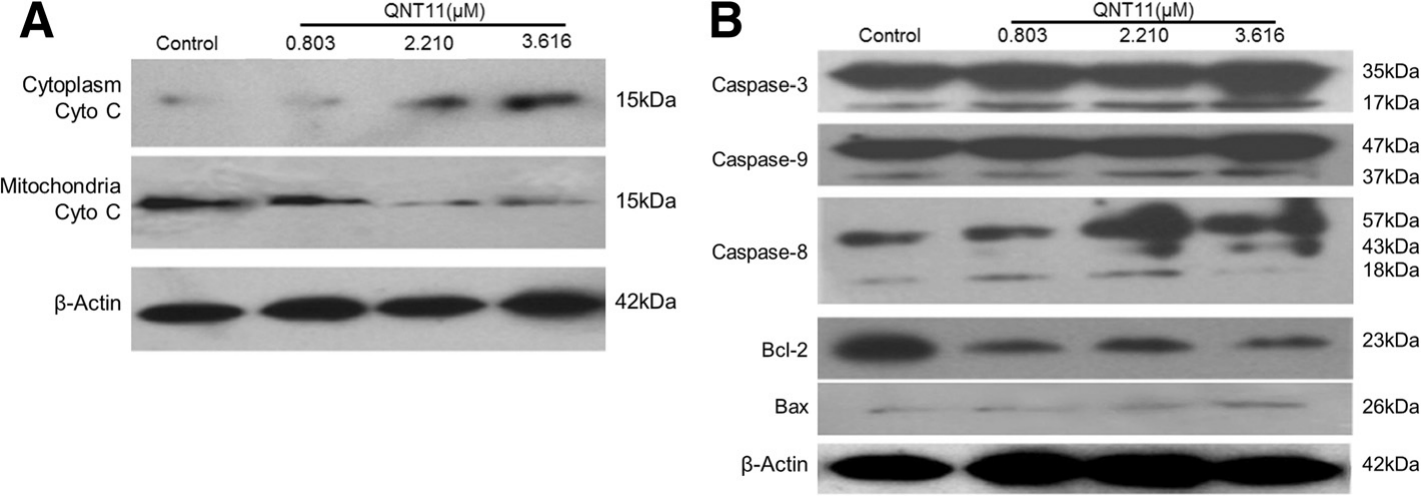
**Effects of QNT11 on apoptotic protein expressions in Hep3B cells. (A)** The Effects of QNT11 on the release of cytochrome c from mitochondria to the cytosol were examined by western blot analysis in Hep3B cells after treatment with 0, 0.803 (IC_30_ group), 2.210 (IC_50_ group) and 3.616 μM (IC_70_ group) of QNT11 respectively for 24 h. **(B)** Protein expressions levels of caspase-9, caspase-8, caspase-3, Bcl-2, and Bax were examined by western blot analysis in Hep3B cells after treatment with 0, 0.803 (IC_30_ group), 2.210 (IC_50_ group) and 3.616 μM (IC_70_ group) QNT11 respectively for 24 h.

The release of cytochrome c from mitochondria is a critical step in the apoptotic cascade that can activate downstream caspases. To examine whether QNT11-induced apoptosis in Hep3B cells was associated with the release of cytochrome c from mitochondrial, the levels of cytochrome c in both the cytosolic and mitochondrial fractions were analyzed. The results showed that there was a significant increase of cytochrome c in the cytosol after 24 h treatment with QNT11 and a decrease in the mitochondrial fraction (Figure 6A).

To characterize the signaling pathways involved in QNT11-induced apoptosis, the expression levels of Bcl-2, Bax,caspase-9, caspase-8, and caspase-3 in QNT11-treated Hep3B cells were analyzed by Western blot analysis (Figure 6B). The anti-apoptotic protein Bcl-2 was decreased, while the pro-apoptotic protein Bax was increased by QNT11 treatment. Because changes in Bax/Bcl-2 levels have been reported in the initiation of caspase signaling, the caspase-9 and caspase-3 activation was examined in this study as well. Upon apoptotic stimulation, full length caspase-9 and caspase-3 were cleaved into active fragments. The results showed that the cleaved activated forms of caspase-9 and caspase-3 increased significantly by 2.210 μM and 3.616 μM of QNT11 treatment. These results indicated that QNT11 induced apoptosis in Hep3B cells through the intrinsic mitochondrial apoptotic pathway. On the other hand, QNT11 significantly increased the expression levels and the cleaved activated forms of caspase-8. Caspase-8 is a prominent initiator of death receptors and is activated by death receptor apoptosis stimuli. Therefore, QNT11 treatment induces apoptotic death in Hep3B cells, at least in part through death-receptor signaling pathway.

### Effects of QNT11 on cell cycle distribution in Hep3B cells

**Figure 7:**
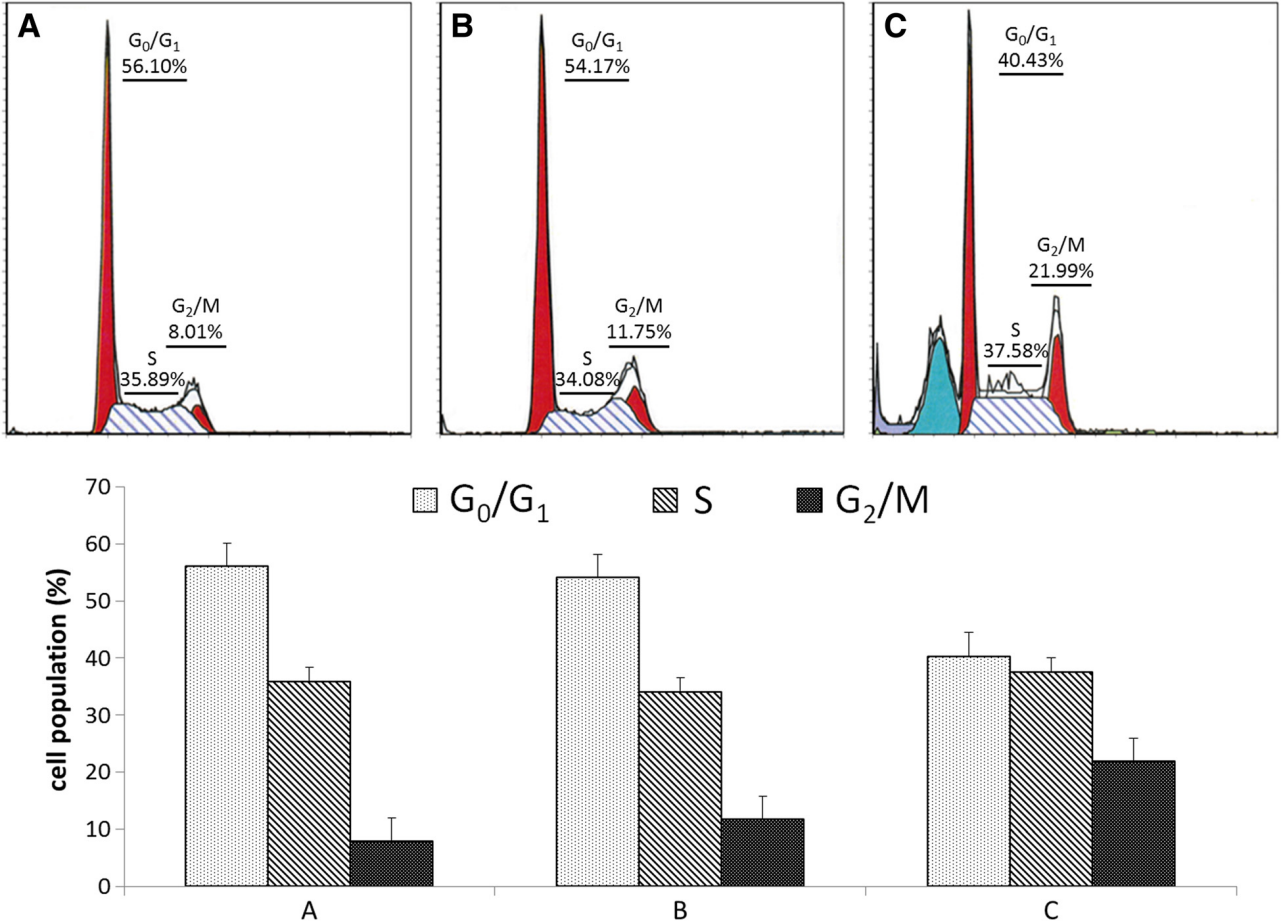
**Effect of QNT11 on the cell cycle distribution in Hep3B cells.** Cell cycle distribution was demonstrated by Flow cytometry analysis in Hep3B cells after treatment with 0 **(A)**, 0.803 **(B)** and 3.616 μM **(C)** QNT11 respectively for 24 h.

As topoisomerase II inhibitors, fluoroquinolones might increase the steady-state concentration of their covalent DNA cleavage complexes and induce cell cycle arrest in the G_2_/M phase and apoptosis. Flow cytometry and propidium iodide was employed to demonstrate the effects of QNT11 on cell cycle distribution and possible apoptosis induction. The sub-G_1_ peak and the accumulation of cells in the G_2_/M phase accompanying reduction in the G_0_/G_1_ phase were observed after the Hep3B cells treatment with 3.616 μM QNT11, which suggested that QNT11 arrested cells in G_2_/M phases and that this arrest might result in sub-G_1_ formation (Figure 7).

**Figure 8:**
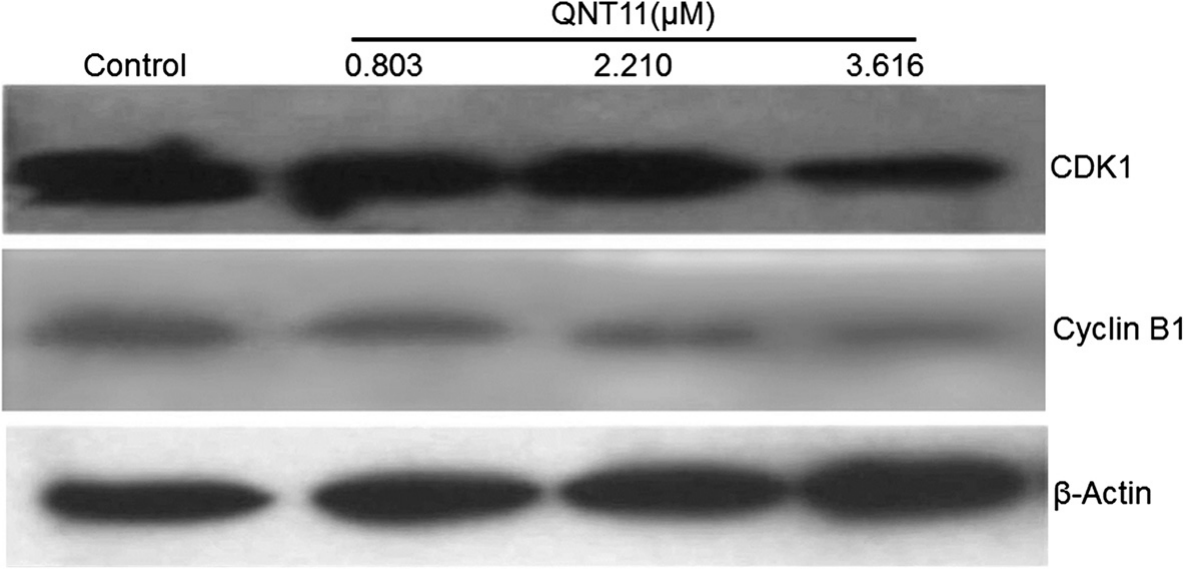
**Effects of QNT11 on CDK1 and cyclin B1 protein expressions in Hep3B cells.** Protein expressions levels of CDK1and cyclin B1 were examined by western blot analysis in Hep3B cells after treatment with 0, 0.803 (IC_30_ group), 2.210 (IC_50_ group) and 3.616 μM (IC_70_ group) of QNT11 respectively for 24 h.

Cyclin B1and CDK1, the master regulators in cell proliferation, plays an essential role in G_2_/M transition of mitosis in cell proliferation. As shown in Figure 8, the expression of CDK1 and cyclin B1 was significantly decreased by the treatment of QNT11 compared with control cells. The result indicated that QNT11 induces G_2_/M arrest by the inhibition of cyclin B/cdk1 complex formation.

## Discussion

Fluoroquinolone compounds have been reported to have an inhibitory effect on cell proliferation and induce apoptosis in carcinoma cell lines [15,16]. In this study, MTT assays indicated that QNT11 inhibited the proliferation of human hepatocarcinoma cells in a dose- and time-dependent manner but did not appear to disturb the proliferation of non-cancerous BMSCs. The result provided the evidence at QNT11 selectively suppresses cancer cell proliferation. Thus, we propose that QNT11 could be an effective candidate for therapy against malignant tumors. Antibacterial fluoroquinolones are a class of antibacterial agents that are commonly used to treat human and animal infections. The treatment of bacterial infection inhibits bacterial DNA gyrase by a mechanism similar to that of certain antitumor drugs against mammalian topoisomerase II [17]. Some antibacterial fluoroquinolones, such as ciprofloxacin, ofloxacin and norfloxacin, also demonstrate a slight interaction with mammalian topoisomerase II, although these antibacterials are much more selective for bacterial DNA gyrase [18]. However, a number of chemically modifed antibacterial fuoroquinolone derivatives with enhanced activity against mammalian topoisomerase II have been developed. Strong inhibitory effects against eukaryotic DNA replication were demonstrated, and their structure-activity relationship has also been characterized [19]. These fluoroquinolone derivatives share a similar mechanism of action with several clinically relevant antitumor agents, such as ellipticine and etoposide. They bind to the topoisomerase II–DNA cleavage complexes, thus converting topoisomerase II into a physiological toxin that creates protein-linked DNA breaks in the genome of treated cells [20]. As shown in Figure 4, QNT11 increased topoisomerase II-mediated supercoiled pBR322 DNA breaks but inhibited topoisomerase II-mediated DNA religation. The effects of QNT11 on the topoisomerase II-mediated DNA cleavage/religation were similar to that of etoposide. These findings provide evidence that QNT11 is a poisonous inhibitor for topoisomerase II [21]. QNT11 binds the reversible complex between DNA and topoisomerase II, preventing the dissociation of the DNA–topoisomerase II complex and thereby inducing DNA damage. It has been reported that the key responses of fuoroquinolone-induced DNA damage is causing cell-cycle arrest and apoptosis of the treated cells [22,23]. Previous studies have shown that fuoroquinolone compounds induced G_2_/M cell cycle arrest and apoptosis in a variety of carcinoma cell lines as well. Cyclin B1 plays an essential role in G_2_/M transition of mitosis in cell proliferation [24,25]. The results of this study showed that the treatment with QNT11 significantly decreased the expression of cyclin B1/CDK1 and confirmed the result of previous study that the effects of fuoroquinolone compounds on cyclin B1 down-regulation, G_2_/M cell cycle arrest and induction of apoptosis.

Apoptosis may be initiated by the stimulation of death receptors located on the cell surface or through an intrinsic pathway involving the release of apoptotic signals from mitochondria [26,27]. The cascading activation of caspases and the release of cytochrome c from the mitochondria play key roles in apoptosis, and the type of intracellular apoptotic pathways involved may be deduced from the activated initiator caspases. We specifically investigated the mitochondria-related events during apoptosis, such as the breakdown of the mitochondrial membrane, the expression of Bax and Bcl-2, and the activation of caspase-9. Members of the Bcl-2 protein family play an important role in apoptosis by regulating the release of cytochrome c from the mitochondria to the cytosol [28]. It has been shown that anti-apoptotic proteins, such as Bcl-2 and Bcl-XL, inhibit cytochrome c release whereas pro-apoptotic members, such as Bax, promote cytochrome c release, leading to the initiation of apoptosis. Here, we observe that QNT11 mediated an up-regulation of Bax and down-regulation of Bcl-2 to induce apoptosis, possibly through increased caspase activity and by preventing the formation of anti-apoptotic bodies. Therefore, it is possible that QNT11 induced the opening of the mitochondrial permeability transition pore through the up-regulation of Bax, resulting in the release of cytochrome c [29,30]. In fact, we observed a QNT11-induced decrease of the mitochondrial membrane potential in Hep3B cells, followed by increased cytochrome c release from the mitochondria into the cytosol. In the mitochondrial apoptotic pathway, the release of cytochrome c is a critical event because cytochrome c forms a complex with procaspase-9 in the cytoplasm (resulting in the activation of procaspase-9), which will eventually lead to the activation of caspases-3 and the induction of apoptosis [31]. Because caspase-8, when activated by the death receptor, is able to cleave the proapoptotic Bcl-2 family member and then trigger a distinct apoptotic pathway involving mitochondria in some fuoroquinolone compound-treated cell types, it is possible that the activation of caspase-9 in QNT11-treated Hep3B cells may be due to the activation of the death receptor-caspase-8 pathway [32,33]. Our results showed that the cleavage of caspase-8 was increased significantly in QNT11-treated cells. Therefore, it is likely that the activation of caspase-9 is triggered mainly by the caspase-8 pathway. However, this needs further evaluation by using caspase inhibitors [34].

## Conclusions

Taken together, we conclude that QNT11 selectively suppressed cancerous cells proliferation through the mechanism of eukaryotic topoisomerase II poisoning. The growth inhibition was in large part mediated via apoptosis-associated mitochondrial dysfunction and regulation of Bcl-2 signaling pathways. The subsequent activation of caspase cascades plays a critical role in QNT11-induced apoptosis in human hepatocarcinoma Hep3B cells. QNT11 may, therefore, have a potential use as a novel chemotherapeutic agent in the treatment of liver cancer, as well as other solid cancers.

## Abbreviations

BMSCs: Bone marrow mesenchymal stem cells; MTT: 3-(4, 5-Dimethylthiazol-2-yl) 2, 5-diphenyltetrazolium bromide; TUNEL: Terminal deoxynucleotidyl transferase-mediated dUTP nick-end labeling; DMSO: Dimethysulfoxide; Bcl-2: B-cell lymphoma 2; Bax: Bcl-2–associated X protein; CDK: Cyclin-dependent kinases.

## Competing interests

The authors declare that they have no competing interests.

## Authors’ contributions

GQH, YHK and BL designed research; JPS, ZYS and SML performed the experiments and data analysis; JBD and CSHF contributed new reagents and analytic tools; JPS, ZYS and BL wrote the paper. All authors read and approved the final manuscript.
